# A 5′- Regulatory Region and Two Coding Region Polymorphisms Modulate Promoter Activity and Gene Expression of the Growth Suppressor Gene *ZBED6* in Cattle

**DOI:** 10.1371/journal.pone.0079744

**Published:** 2013-11-06

**Authors:** Yong-Zhen Huang, Ming-Xun Li, Jing Wang, Zhao-Yang Zhan, Yu-Jia Sun, Jia-Jie Sun, Cong-Jun Li, Xian-Yong Lan, Chu-Zhao Lei, Chun-Lei Zhang, Hong Chen

**Affiliations:** 1 College of Animal Science and Technology, Northwest A&F University, Shaanxi Key Laboratory of Molecular Biology for Agriculture, Yangling, Shaanxi, People’s Republic of China; 2 United States Department of Agriculture, Agricultural Research Service, Bovine Functional Genomics Laboratory, Beltsville, Maryland, United States of America; 3 Institute of Cellular and Molecular Biology, Jiangsu Normal University, Xuzhou, Jiangsu, People’s Republic of China; CSIRO, Australia

## Abstract

*Zinc finger, BED-type containing 6* (*ZBED6*) is an important transcription factor in placental mammals, affecting development, cell proliferation and growth. Polymorphisms in its promoter and coding regions are likely to impact *ZBED6* transcription and growth traits. In this study, rapid amplification of 5’ cDNA ends (5'-RACE) analysis revealed two transcription start sites (TSS) for the bovine *ZBED6* starting within exon 1 of the *ZC3H11A* gene (TSS-1) and upstream of the translation start codon of the *ZBED6* gene (TSS-2). There was one SNP in the promoter and two missense mutations in the coding region of the bovine *ZBED6* by sequencing of the pooled DNA samples (Pool-Seq, n = 100). The promoter and coding region are the key regions for gene function; polymorphisms in these regions can alter gene expression. Quantitative real-time PCR (qPCR) analysis showed that *ZBED6* has a broad tissue distribution in cattle and is highly expressed in skeletal muscle. Eleven promoter-detection vectors were constructed, which enabled the cloning of putative promoter sequences and analysis of *ZBED6* transcriptional activity by luciferase reporter gene assays. The core region of the basal promoter of bovine *ZBED6* is located within region -866 to -556. The activity of WT-826G-pGL3 in driving reporter gene transcription is significantly higher than that of the M-826A-pGL3 construct (*P* < 0.01). Analysis of gene expression patterns in homozygous full-sibling Chinese Qinchuan cattle showed that the mutant-type Hap-AGG exhibited a lower mRNA level than the wild-type Hap-GCA (*P* < 0.05) in longissimus dorsi muscle (LDM). Moreover, *ZBED6* mRNA expression was low in C2C12 cells overexpressing the mutant-type *ZBED6* (pcDNA3.1^+^-Hap-GG) (*P* < 0.01). Our results suggest that the polymorphisms in the promoter and coding regions may modulate the promoter activity and gene expression of bovine *ZBED6* in the skeletal muscles of these cattle breeds.

## Introduction

A quantitative trait locus (QTL) is a genomic region that affects a quantitative trait or traits that vary in degree and can be controlled by multiple loci [1]. Most economically important traits of interest in cattle, such as growth, carcass, fatness and meat quality, have a multifactorial background and are controlled by environmental factors and an unknown number of QTLs. These quantitative traits arise from interactions between two or more genes and their environment and can be mapped to their underlying genes via closely linked stretches of DNA. *IGF2* is a secreted peptide hormone that plays an important role in muscle tissues via both endocrine and local autocrine/paracrine mechanisms primarily by stimulating the *IGF1 receptor* (*IGF1R*). The muscle hypoplasia phenotype of *IGF1R*-null mice confirms the necessity of IGF signaling in muscle development [2-4]. A paternally expressed QTL affecting muscle growth, fat deposition and heart size in pigs maps to the insulin-like growth factor 2 (IGF2) region [5,6]. A single nucleotide substitution in intron 3-G3072A of *IGF2* in pigs abrogates a binding site for a repressor and leads to a threefold up-regulation of *IGF2* expression in skeletal muscle [7]. This quantitative trait nucleotide (QTN) is one of the rare examples in which a single base substitution underlying a complex trait has been identified and the mechanism of action is partially understood [7].

*Zinc finger, BED-type containing 6* (*ZBED6*) is a novel transcription factor that was identified and shown to act as a repressor of *IGF2* transcription in skeletal muscle myogenesis and development [8]. ZBED6 has a single exon comprising more than 900 codons and two DNA-binding BED domains. *ZBED* genes originate from domesticated hAT DNA transposons and encode regulatory proteins of diverse function in vertebrates. The number of BED domains varies among ZBED proteins, and the ability of *ZBED6* to interact with chromatin and affect transcriptional regulation is most likely a function derived from the ancestral active transposons. Phylogenetic analyses demonstrate that *ZBED6* form a monophyletic group, which is distinct from the *ZBED1* gene. The alignments of ZBED-derived BED domains suggest multiple independent duplications of sequences encoding BED domains after ZBED gene domestication [9]. ZBED6 is specific for placental mammals and derived from a domesticated DNA transposon [10]. In addition, ZBED6 is a novel transcription factor that appears to have evolved an essential function in the common ancestor of all placental mammals [11]. The *ZBED6* gene is exclusive to placental mammals and highly conserved among species; it is found at the same genomic position and shows near 100% amino acid identity across 26 placental mammals according to the available genome sequence data [8]. An electrophoretic mobility shift assay (EMSA) showed that *ZBED6* is a bona fide repressor of *IGF2* transcription that interacts with the QTN region of *IGF2* [8]. The functional characterization of *ZBED6* shows that it has a broad tissue distribution and may affect the expression of approximately 2,500 putative downstream targets, many of which have essential biological functions in placental mammals [8].

Here, we show the promoter activity and gene expression affected by three single nucleotide substitutions in the *ZBED6* gene. mRNA expression patterns in eight cattle tissues and organs were obtained using qPCR, and we determined the minimal region required for promoter activity within the upstream bovine *ZBED6* gene sequences. Moreover, we assessed whether the variants affect bovine *ZBED6* gene transcription and compared bovine *ZBED6* expression in longissimus dorsi muscle (LDM) from wild-type and mutant-type haplotype animals to clarify that the effect of the variants on *ZBED6* overexpression is associated with *IGF2* expression. Thus, we hypothesized that the *ZBED6* promoter and coding region polymorphisms may modulate gene expression and thereby influence skeletal muscle myogenesis and development. This hypothesis could provide an insight into the transcriptional regulation of the *ZBED6* gene and the potential biological roles of its gene product.

## Materials and Methods

### Ethics Statement

The study protocol was approved by the Regulations for the Administration of Affairs Concerning Experimental Animals (Ministry of Science and Technology, China, 2004) and approved by the Institutional Animal Care and Use Committee (College of Animal Science and Technology, Northwest A&F University, China). Bovine embryos of slaughtered cows (Chinese Qinchuan) were collected from Tumen Abattoir, a local slaughterhouse in Xi'An, China. Newborn Chinese Qinchuan calf and adult Chinese Qinchuan cattle were obtained from Shannxi Kingbull Animal Husbandry Co., Ltd (Baoji, China). Adult animals were allowed access to feed and water *ad libitum* under normal conditions and humanely sacrificed as necessary to ameliorate suffering.

### Samples and Sequencing

To explore the genetic variation of bovine *ZBED6* genes, we sequenced pooled DNA samples (Pool-Seq, n = 100) from four cattle breeds in China: Nanyang cattle (NY), Qinchuan cattle (QC), Jiaxian cattle (JX) and Chinese Holstein (CH). The pooled DNA samples consisted of 25 DNA samples selected randomly from each cattle breed. NY, QC and JX are three important breeds for beef production in China, whereas CH is a dairy breed. These four breeds are the main breeds of China, and they are reared in the provinces of Henan and Shaanxi. Calves were weaned at six months of age on average and raised from weaning to slaughter on a diet of corn and corn silage. The animals of each breed were selected to be unrelated for at least three generations with the aim of having diverse lineages within each breed.

Genomic DNA was isolated from 2% heparin-treated blood samples and stored at -80 °C following standard procedures [12]. The content of DNA was estimated spectrophotometrically, and the genomic DNA was then diluted to 50-100 ng/μl. All DNA samples were stored at -20 °C for subsequent analysis.

Primers used to amplify the bovine *ZBED6* gene (NCBI: AC_000173.1) were designed from a published gene sequence. Primers, fragment sizes and annealing temperature (AT) are provided in Table S1.

### Tissue and Cell RNA Isolation and Quantitative Real-Time PCR (qPCR)

Samples of eight tissues and organs (heart, liver, spleen, lungs, stomach, intestines, longissimus dorsi muscle (LDM) and adipose tissue) from nine individuals (three individuals per stage) were harvested for RNA isolated within 10 min after slaughter at three key stages of myogenesis and muscle maturation: 90 days at embryo (fetal bovine, FB), 3-day-old (newborn bovine, NB) and 24-month-old (adult bovine, AB).

An analysis of gene expression patterns in six homozygous individuals from full-sibling Chinese Qinchuan cattle (i.e., in which all six animals have the same sire and dam) was conducted. The LDM sample was collected from castrated full-sibling individuals (three wild-type Hap-GCC animals and three mutant-type Hap-AGG animals) of the AB raised by Shannxi Kingbull Animal Husbandry Co., Ltd. (Baoji City, China). The specimens used in this study were collected immediately after slaughter and used for gene expression analysis. Muscle specimens for gene expression analysis were sampled and snap frozen in liquid nitrogen. Total RNA was isolated from 80-100 mg of tissue samples from QC, and RNA from C2C12 cell samples was isolated using TRIzol reagent (Invitrogen, Carlsbad, CA, USA) according to the manufacturer’s protocol. All samples were subjected to reverse transcription using a cDNA high capacity kit (Invitrogen, Carlsbad, CA, USA).

The qPCR analysis using SYBR Green PCR Master Mix (Takara, Dalian, China) was performed on a CFX96™ Real-Time PCR Detection System (Bio-Rad, USA). Data were normalized to the geometric mean of data from bovine *GAPDH* and *ACTB*, which were used as endogenous control genes. All primers for validation were designed to cross exon-exon junctions and are shown in Table S2. Relative expression levels of objective mRNAs were calculated using the ∆∆Ct method [13].

### Rapid Amplification of 5’ cDNA ends (5’-RACE)

The SMARTer^TM^ RACE cDNA Amplification kit (Clontech, California, USA) was used to amplify 5’-cDNA ends of bovine skeletal muscle mRNA. First strand cDNA synthesis was carried out according to the manufacturer’s instructions with bovine total RNAs, 5’ CDS primer A, SMART II A oligonucleotide and TITANIUM *Taq* DNA polymerase. The 5’-RACE PCR reaction was performed according to the manufacturer’s protocol using universal primer A mix (UPM, forward primer) and two antisense gene specific primers (GSP) to the known exon of the *ZBED6* gene (GSP1 and GSP2) using the protocols provided with the RACE kit. To verify the RACE products, nested 5’-RACE PCR was carried out using nested UPM (NUPM) forward primer and the nested specific reverse primers (NGSP) of the *ZBED6* gene (NGSP1). The primer sequences are noted in Table S4. Good quality RACE products were separated by 2% agarose gel electrophoresis, and the resulting bands were then extracted from the gel. Each product was ligated into pGEM® T-easy vector (Promega, Madison, WI, USA). All nested RACE products were randomly selected for sequencing. Additional 5’-RACE experiments were carried out using primers closer to the 5’ end of the new sequence to confirm the 5’ end of the mRNA.

### Bioinformatic Analysis of Transcription Factor Binding Sites (TFBSs) in the *ZBED6* Promoter Region

The in silico tool prediction of transcription factor binding sites (TFBSs) in promoter sequences was performed using the Genomatix MatInspector software (http://www.genomatix.de/) with standard settings for the highest matrix similarity [14]. This program uses a large library of weight matrices based on known in vivo binding sites to predict TFBS in nucleotide sequences.

### Plasmids

#### Construction of Deletion Mutants of the ZBED6 Promoter

The genomic DNAs were extracted from the longissimus dorsi muscle tissues of the AB using a phenol extraction method [12]. Twelve different primers (listed in Table S3) designed according to the sequence of the bovine *ZBED6* gene were used in the PCR with bovine genomic DNA as a template to obtain the complete bovine *ZBED6* promoter (P1) and the truncated promoter fragments (P2 to P11).

#### Construction of Promoter Haplotypes of ZBED6

Based on genotyping results, genomic DNA isolated from QC with homozygous GG and AA genotypes was used as a template for constructing plasmids of wild-type haplotype WT-836G-pGL3 and mutant-type haplotype M-826A-pGL3, respectively. A DNA fragment that covers the polymorphic sites (SNP1: *ZBED6*-Promoter -826G>A) ranging from -866 to +25 (relative to initiation codon ATG) of the 5’-regulatory region of the bovine *ZBED6* gene was amplified by PCR with PrimeSTAR HS DNA polymerase (2.5 U/μL) (Takara, Dalian, China) and ligated into the pGL3-basic vector (Promega, Madison, WI, USA). The primer used for constructing vectors WT-836G-pGL3 and M-826A-pGL3, which contains *Kpn* I (forward primer) and *Bgl* II (reverse primer) restriction sites, is indicated by the lowercase letters in Table S3.

After being digested by restriction enzymes *Kpn* I and *Bgl* II, PCR products were separately inserted into the pGL3-base vector (Promega, Madison, WI, USA), which contains the luciferase reporter gene, according to the manufacturer’s instructions.

#### Construction of the pcDNA3.1^+^-ZBED6 Expression Vector

The primer pairs (Table S2) designed according to the GenBank sequence database were used in PCR to obtain the CDS sequence of the bovine *ZBED6* gene. The restriction enzymes *Kpn* I (forward primer) and *Xba* I (reverse primer) were used to digest the amplified bovine *ZBED6* CDS and pcDNA3.1^+^ vector (Invitrogen, Carlsbad, CA, USA). T4 DNA ligase (Takara, Dalian, China) was used to link the products of digestion. Standard molecular cloning techniques [12] were then used to obtain the pcDNA3.1^+^- *ZBED6* vectors.

### Cell Culture and Treatments

Mouse myoblast cell line C2C12 was purchased from American Type Culture Collection. C2C12 cells were maintained in Dulbecco's modified Eagle's medium (DMEM) supplemented with 10% fetal bovine serum (FBS), 100 units/ml penicillin (final concentration) and 100 μg/ml streptomycin. Cells were cultured in a 5% CO_2_ humidified incubator at 37 °C.

### Plasmid Transfection and Dual Luciferase Reporter Assay

When cells reached 90% confluence, the proliferation medium was removed, and the cells were rinsed with phosphate-buffered saline and treated for 2 min with 0.25% trypsin to detach cells. Cells were collected, centrifuged and diluted in a proliferation medium prepared with DMEM without penicillin/streptomycin (pen/strep) and split onto two gelatin-coated 24-well plates at a density of ~10^5^ cells per well. The following day, cells were transfected with Lipofectamine 2000 (Invitrogen, Carlsbad, CA, USA) according to the manufacturer’s instructions. Briefly, for each well, 2.0 μl of Lipofectamine 2000 and 0.8 μg of DNA were mixed in 100 μl of FBS-free and pen/strep-free Opti-MEM I medium (Promega, Madison, WI, USA) for 20 min. To normalize the transfection efficiency, the pRL-TK plasmid vector (Promega, Madison, WI, USA), which carries a renilla luciferase gene, was co-transfected with the reporter construct as described above. The experiments were performed in three replicates for each construct.

The relative activities of the full-length promoter and the truncated promoter fragments of the bovine *ZBED6* gene were analyzed using the Dual Luciferase Reporter Assay System (Promega, Madison, WI, USA) in accordance with the manufacturer’s protocol. Cells were harvested 24 h post-transfection, and firefly and renilla luciferase activities were measured using the Dual-Luciferase Reporter Assay System (Promega, Madison, WI, USA) and a BHP9504 Fluorescent Analytic Instrument (Hamamatsu, Japan). The firefly luciferase activities were normalized by the renilla luciferase activities in each well. The data given in the results section are the average of three replicates. Data are expressed as the mean ± standard error from two independent experiments.

## Results

### Variants Discovery

We amplified and sequenced the *ZBED6* gene from 100 DNA samples selected randomly from the study cattle population. Compared with the published gene sequence from NCBI, there were three SNPs identified in these animals, including one non-coding mutation in the promoter (SNP1: -826G>A) and two coding mutations in the CDS (SNP2: 680C>G and SNP3: 1043A>G) of the *ZBED6* gene. The SNP2 and -3 are missense mutations: p. Ala 227 Gly and p. His 348 Arg. The promoter SNP was novel and submitted to the NCBI dbSNP (Table 1).

**Table 1:** Summary of the SNPs in the bovine *ZBED6* gene.

**SNPs**	**Variant location**	**Variant type^1^**	**Alleles mutation**	**Amino acid change**	**DPS (nt)^2^**	**No** ^3^ **.**
1	Promoter	-826G>A	AGG/AGA	non-coding	0	ss#647298822
2	Exon 1	680C>G	GCA/GGA	Ala 227 Gly	1506	rs110267555
3	Exon 1	1043A>G	CAT/CGT	His 348 Arg	2549	rs133141901

^1^ The location is indicated to the upstream or downstream of the translation initiation ATG codon.

^2^ DPS: Distance from previous SNP (nt).

^3^ No.: Single Nucleotide Polymorphism database (dbSNP) number.

### Expression Profile

**Figure 1:**
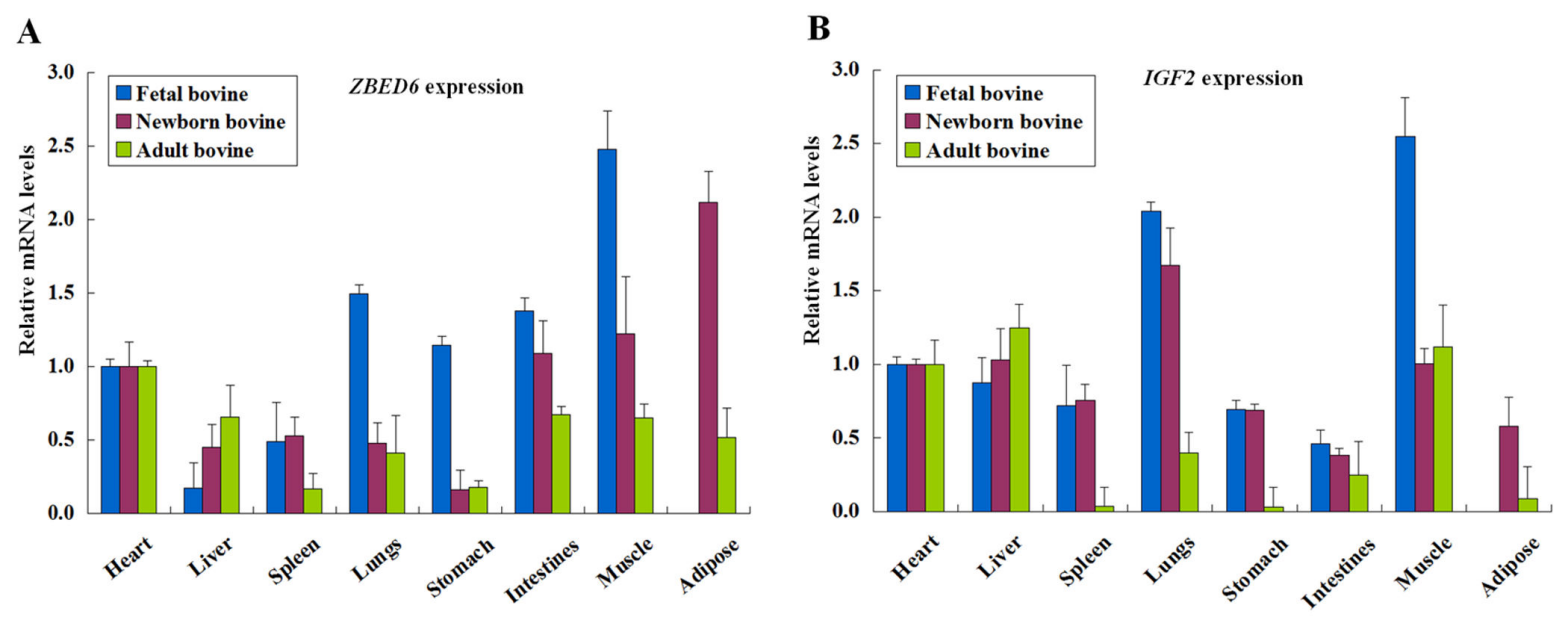
Expression pattern analysis of *ZBED6* and *IGF2* in several bovine tissues and organs. *ZBED6* (A) and *IGF2* (B) mRNA expression were normalized to the geometric mean of the two suitable housekeeping genes (*GAPDH* and *ACTB*) and expressed relative to gene expression in the hearth group. Error bars represent standard error of the mean (SE). Each column value represents the means±SE of three replicates.

To detect the tissue distribution of bovine *ZBED6* and *IGF2* mRNA, qPCR was carried out with cDNA from eight cattle tissues and organs (heart, liver, spleen, lungs, stomach, intestines, LDM and adipose tissue). As shown in Figure 1, the qPCR analysis showed that *ZBED6* (Figure 1A), similar to *IGF2* (Figure 1B), has a broad tissue distribution in cattle tissues and organs, which is consistent with its role as the *IGF2* repressor in all examined tissues and organs from nine individuals (three individuals per stage). The expression patterns of the *ZBED6* and *IGF2* genes were similar; the relative expression levels were decreased in all tissues except liver from day 90 bovine embryos (fetal bovine, FB) to 24-month-old adults (adult bovine, AB), respectively. Specifically, in the fetal stage, *ZBED6* and *IGF2* had the highest expression levels in the muscle tissues. In newborn stage, *ZBED6* and *IGF2* had the highest expression levels in the adipose and lung tissues, respectively. In adult stage, *ZBED6* and *IGF2* had the highest expression levels in the heart and liver, respectively.

### Transcription Start Site (TSS) Mapping

To verify the location of the TSS in the *ZBED6* gene, we performed 5’-RACE experiments on bovine skeletal muscle total RNA. Three reverse gene-specific primers were designed for the PCR step that follows cDNA synthesis in the 5’-RACE process. Primers GSP1 and GSP2 anneal just downstream of the TSS and the exon of the *ZBED6* gene.

**Figure 2:**
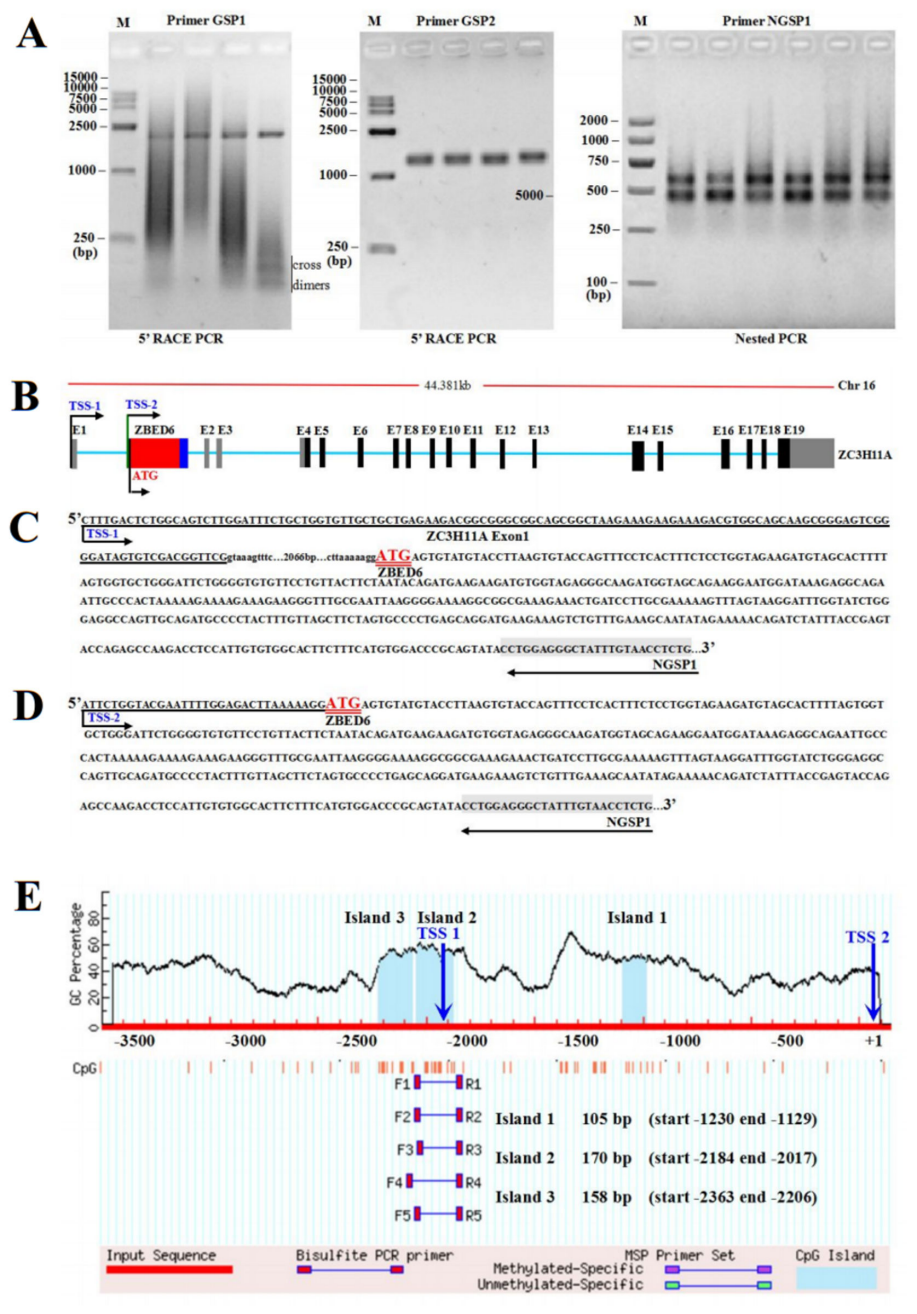
Identification of the transcription start site of *ZBED6* by rapid amplification of cDNA 5’-ends (5’-RACE). (A) 5 ’-RACE reactions were performed on cDNA synthesized from skeletal muscle mRNA with primers specific for *ZBED6*. Representative ethidium bromide-stained agarose gels of PCR products are shown. A 5’-RACE CDS primer (Stratagene) and two *ZBED6* gene-specific primers (GSPs) were used with expected product sizes: GSP1 (>2042 bp, from ATG to 3’ end of GSP1) and GSP2 (>1351 bp, from ATG to 3’ end of GSP2). The 5’-RACE product from each reaction was used as template in a subsequent nested PCR reaction. The expected sizes of the nested PCR products were greater than 584 bp and 489 bp (from ATG to 3’ end of NGSP1). (B) Genome organization of *ZBED6* and *ZC3H11A* in cattle. Untranslated and translated exons of *ZBED6* are indicated by blue and red bars, respectively. Untranslated and translated exons of *ZC3H11A* are indicated by gray and black bars, respectively. (C) and (D) Sequence of the 5’ untranslated region of the bovine *ZBED6*. The positions of transcriptional start sites (TSS) and the start codon (ATG) as defined by 5’-RACE. The translational start site (ATG) is shown by a double underline. 5’ untranslated and exon sequences are shown in capitals, and introns are shown in small letters with the total intron sizes shown. The nucleotides identified by sequencing the cDNA clones obtained from 5’-RACE are underlined. (E) Schematic representation of the proximal promoter region (+1 to -3640 base pairs) of the bovine *ZBED6* gene (modified output of MethPrimer program [32]) to predict regions of high GC content. Dashed lines indicate the GC percentage as represented on the y-axis and the x-axis denotes the bp position on the *ZBED6* gene 5’ untranslated region. Arrows indicate the TSS. Coordinates are given in relation to the translation initiation site (shown as +1); vertical lines indicate relative positions of CpG dinucleotides; solid lines depict location of the PCR primers. The 105 bp, 170 bp, and 158 bp CpG islands are evident in the 5' part of the gene, with GC content of 51.96%, 54.17%, and 55.70%.

The 5’-RACE PCR on bovine total RNA was used as a template together with primers GSP1 and GSP2. The bands that were visible at approximately 2.2 kb and 1.5 kb were close to the expected product size (Figure 2A), and there were some faint bands of low molecular weight (<250 bp) of the cross-dimer products between the GSP1 and 5’-RACE UPM primers (as verified by sequencing) (Figure 2A).

To increase specificity and sensitivity, a second round of PCR was performed using nested primers on the products of the 5’ -RACE PCR. The expected amplification sizes with the NCBI-predicted TSS were 584 bp and 461 bp for NGSP1 (Figure 2A). Sequence analysis revealed a partial alignment of the 584 bp (TSS-1) and 495 bp (TSS-2) NGSP1 product, with *ZBED6* starting within exon 1 of the *ZC3H11A* gene (TSS-1) and upstream of the ATG of the *ZBED6* gene (TSS-2) (Figure 2B and 2C). Analysis of these sequences demonstrated that the products corresponded to two distinct transcripts starting at -123 and -32 with respect to the *ZBED6* translation start site, which will be referred to as +1 throughout this report (Figure 2C; 2D). Conventionally, transcriptional initiation is believed to occur from a single focused “TSS” [16]. Previous experiments have suggested that *ZBED6* is an intronless gene located in the first intron of *ZC3H11A*, and the *ZBED6* gene is co-transcribed with *ZC3H11A* by using a common promoter [8]. When the *ZC3H11A* first intron containing *ZBED6* is present in the transcript, only ZBED6 protein is translated because it contains the first translated codon and a termination codon. Transcripts of the *ZC3H11A* gene will be translated into proteins only when the first intron is spliced out [8,11]. Therefore, the presence of two distinct transcriptional start sites suggests that *ZBED6* transcription may be regulated by “non-traditional” mechanisms.

### Bioinformatic Analyses Predict the Allele-Dependent Presence of Different TFBS in the *ZBED6* Promoter Region

**Figure 3:**
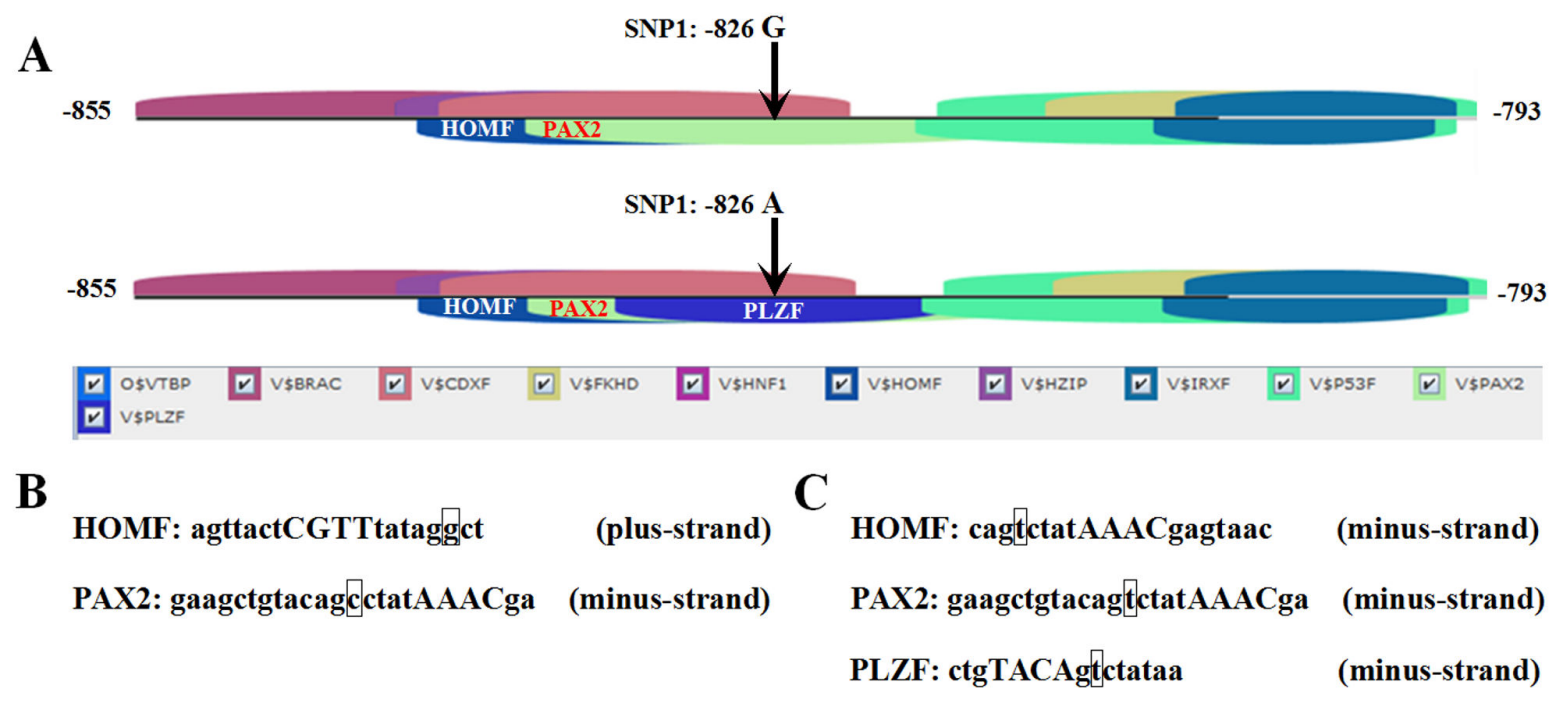
Schematic presentation of the location of analyzed polymorphisms in the bovine *ZBED6* gene promoter and surrounding TFBS modified from Genomatix MatInspector software output. (**A**) A PLZF binding site is predicted when the major “A” allele of the SNP1 (-826G>A) is present but lacking when the “G” allele of the SNP1 (-826G>A) is present. (**B**) and (**C**) Sequence that was recognized by Genomatix MatInspector to contain a TFBS and also the “A” allele of the SNP1 (B) or the “G” allele of the SNP1 (C). Nucleotides in capitals denote the core sequence used by MatInspector. Square box nucleotides highlight the position of the respective polymorphism. The plus-strand indicates that a TFBS sequence was found for the positive strand, and the minus-strand indicates that a TFBS sequence was found for the opposite strand.

Bioinformatic analyses using the Genomatix software predicted transcription factors with the highest core matrix similarities and revealed 481 *cis*-acting elements in the 1.5 kb region (5’ relative to the translation start site) of the wild-type (Table S5) and mutant-type (Table S6) *ZBED6* promoter region. Accordingly, a total of 82 acting elements were scored on both strands of the promoter core sequence (-866 to -556), of which 44 were located in the plus strand and 38 in the minus strand. Several binding sites for transcription factors were predicted for the region containing SNP1 (-826G>A); the *homeobox gene family -1* (*HOMF*), *paired box gene 2* (*PAX2*) and promyelocytic leukemia zinc finger (PLZF) directly span the 826 position. Interestingly, the *PLZF* binding site is present only when the *ZBED6* promoter sequences contain the 826 major “A” allele, because the 826 major “A” allele is part of the binding site core sequence of *PLZF* (Figure 3A, B and C).

### Promoter Analysis of the *ZBED6*

**Figure 4:**
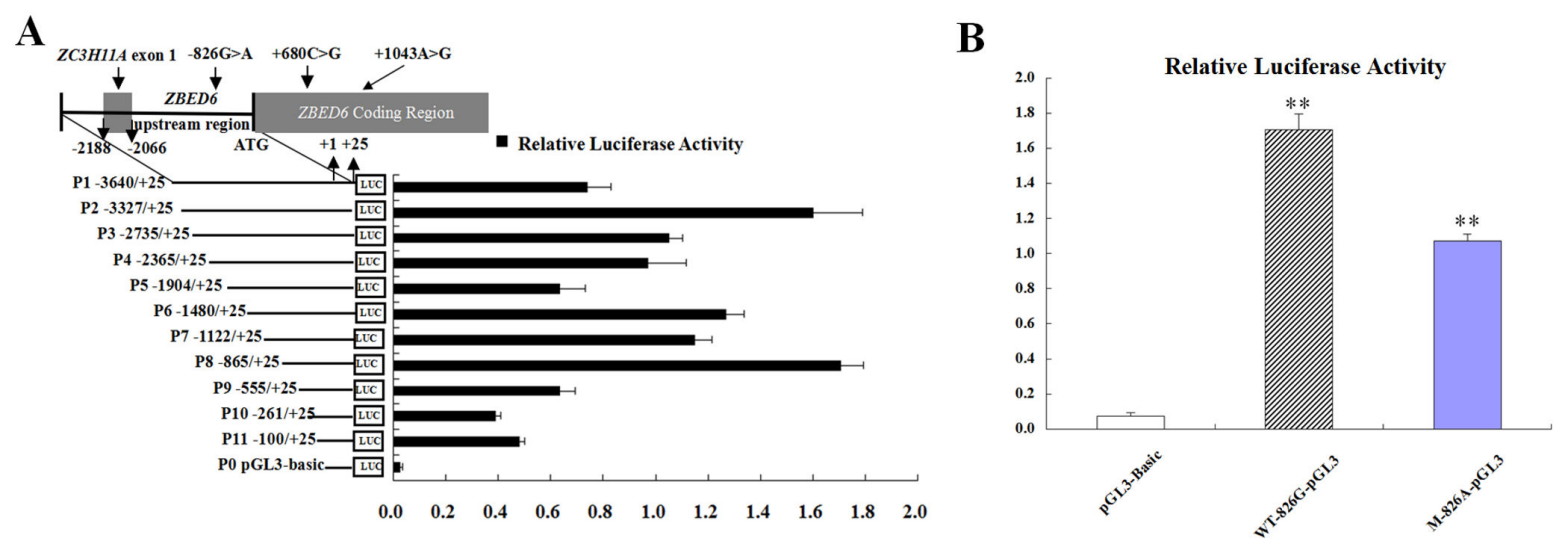
Recombinant vectors used to assay the upstream sequence activity of *ZBED6* and *ZC3H11A*. (**A**) Luciferase activity of the *ZBED6* (black bars) and *ZC3H11A* (gray bars) upstream sequences deletion constructs in C2C12 cells. The location and size of each 5’-deletion fragment is indicated to the left of each bar relative to the translation initiation ATG codon. (**B**) *ZBED6* promoter activity modulated by genetic polymorphisms. Luciferase activity was transfected with recombinant plasmids containing the *ZBED6* 5’ region haplotypes (826G and 826A) of SNP1 (ZBED6 promoter -826G>A) in cell lines. Results from pGL3-Basic plasmid (Open bars) are given as a negative control. Diagonal bars show luciferase activities from cell lines transfected with the wild type haplotype WT-826G-pGL3 construct containing the wild-type allele. Thick solid bars represent luciferase activities from cell lines transfected with the mutant haplotype M-826A-pGL3 construct containing the mutant allele. Data are mean ± SE of normalized luciferase activity. (Values represent the mean ± SE of three duplications.) *P <0.05, **P <0.01.

We next sought to determine the minimal region required for promoter activity within the upstream bovine *ZBED6* gene sequences. A series of deletion constructs from the 5’-flank region of this gene was generated via PCR-based approaches (Figure 4A). These deletion constructs were transiently transfected into C2C12 cells, and luciferase activity was detected. The results showed that deletion constructs P1 to P6 displayed elevated promoter activity relative to P0 (Figure 4A). They indicated a potential enhancer located between nucleotides -101 and +25 because this region was adjacent to the translation initiation codon (ATG) and far away from the TSS [15]. Moreover, deletion construct P8 (nucleotides -866 to +25) had significantly elevated promoter activity relative to others, leading us to conclude that the core region of the basal promoter of bovine *ZBED6* is located within the -866 to -556 sequence upstream of *ZBED6*, which is a region in the vicinity of the TSS1 (Figure 4A).

By generating a series of deletion constructs of the bovine *ZBED6* upstream region, the optimal promoter was defined to be the 311 bp (-866 to -556) region upstream of the initiation codon. Sequence analysis of this region showed that it lacked a TATA box, which is known to recruit transcriptional machinery needed for efficient expression of a gene [16]. Recent bioinformatics studies have suggested that the majority of mammalian gene promoters lack a “TATA box”, have multiple TSSs and are highly GC-rich [17-20]. Our 5’-RACE analysis demonstrated that the *ZBED6* upstream region contained two transcriptional start sites (Figure 2A) and had a significantly high GC content in the three CpG islands (51.96%, 54.17% and 55.70%.) (Figure 2E).

### Comparison of the Promoter Region of *ZBED6* Harboring Different Haplotypes in Driving Transcription

To assess whether the haplotypes affect bovine *ZBED6* gene transcription, the 896 bp fragment spanning the 5’-flanking region from -1481 to +25 (relative to initiation codon ATG) was cloned and linked to a luciferase reporter construct. Two constructs differing only at promoter -826 were obtained, both of which harbor the identified haplotypes, namely the wild-type haplotype WT-826G-pGL3 and the mutant-type haplotype M-826A-pGL3. The promoter activity of these haplotypes was assessed by transient transfection of the individual reporter constructs into C2C12 cells. As shown in Figure 4B, the activity of WT-826G-pGL3 in driving reporter gene transcription is significantly higher than that of the M-826A-pGL3 construct (*P* < 0.01). Of the two haplotypes identified in C2C12 cells, WT-826G-pGL3 has the highest activity, and M-826A-pGL3 has the lowest activity.

### Comparison of mRNA Expression in LDM from Homozygous Wild-Type and Mutant-Type Animals

**Figure 5:**
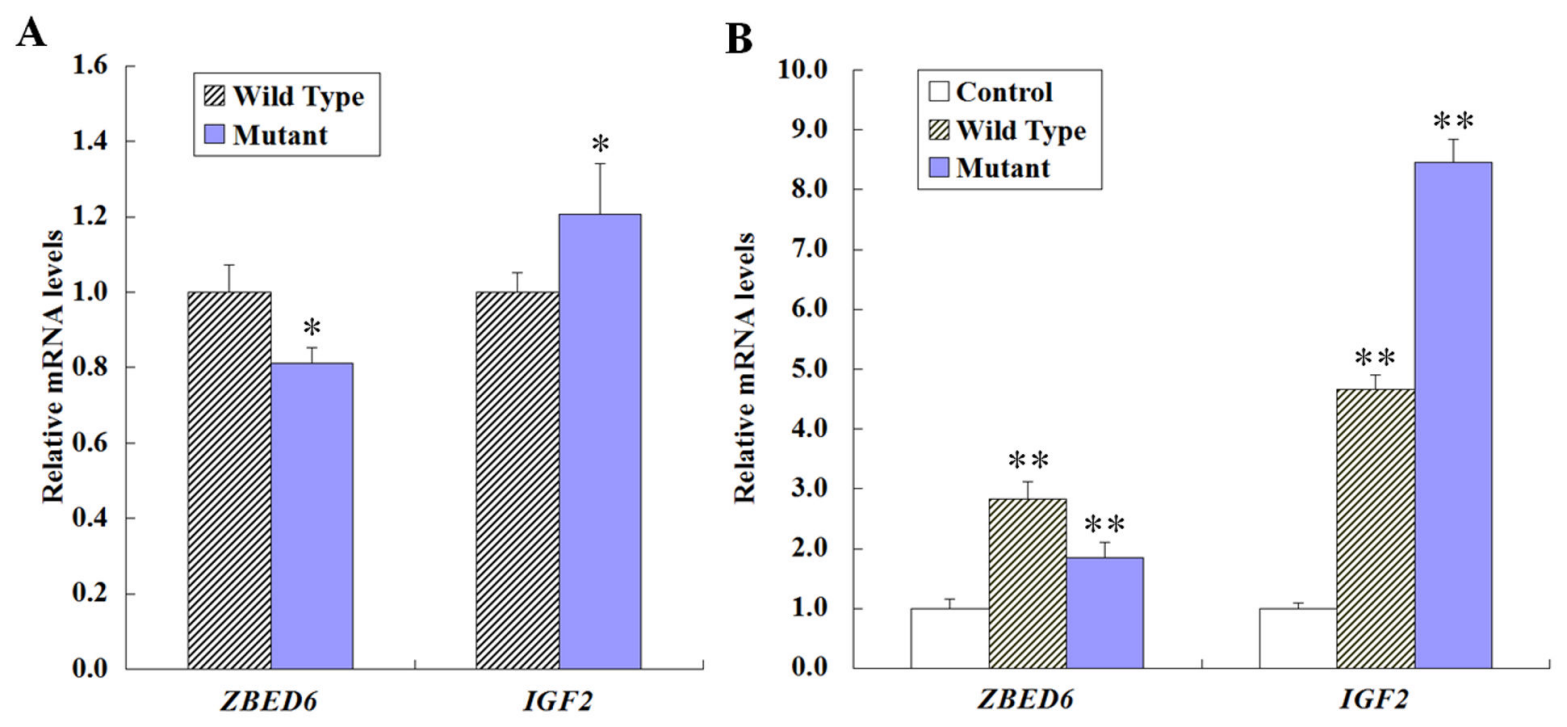
Expression pattern of *ZBED6* and *IGF2* in longissimus dorsi muscle (LDM) and C2C12 cell lines. The mRNA expression was normalized to the geometric mean of the two suitable housekeeping genes (*GAPDH* and *ACTB*) and expressed relative to gene expression in the control group. Error bars represent standard error of the mean (SE). Each column value represents the mean±SEM of three replicates. (A) Comparison of mRNA expression levels of bovine *ZBED6* and *IGF2* in LDM between homozygous wild-type haplotype GCA (Hap-GCA, n = 3) and homozygous mutant haplotype AGG (Hap-AGG, n =3). (B) qPCR analysis of *ZBED6* and *IGF2* mRNA levels in C2C12 cells transfected with pcDNA3.1^+^ (control), pcDNA3.1^+^-Hap-CA (wild-type) or pcDNA3.1^+^-Hap-GG (mutant-type). A blank expression vector (pcDNA3.1^+^) was used to maintain equivalent amounts of DNA. Data are presented as the means±SE of at least three independent experiments. *P < 0.05, **P < 0.01.

As described above, an in vitro study indicated that compared with WT-826G-pGL3, M-826A-pGL3 has lower activity in driving reporter gene transcription. The bovine *ZBED6* and *IGF2* mRNA expression in LDM was compared between full-sibling cattle (share the same sire and dam) carrying the homozygous wild-type haplotype GCA (Hap-GCA, n = 3) and the homozygous mutant-type haplotype AGG (Hap-AGG, n = 3). The results indicated that the bovine *ZBED6* mRNA level was lower in mutant-type Hap-AGG (0.8123±0.041) than in wild-type Hap-GCA (1.0000±0.072) in LDM (*P* < 0.05); however, the mutant-type Hap-AGG (1.2058±0.135) exhibited a higher *IGF2* mRNA level than the wild-type Hap-GCA (1.0000±0.052) (*P* < 0.05) (Figure 5A). This result suggested that *ZBED6* acts as a loss-of-function mutation in the homozygous mutant-type individuals and that the *ZBED6* mutation alleles lead to up-regulation of *IGF2* expression in the skeletal muscle of cattle.

### Over-expression of *ZBED6* Is Associated with *IGF2* Expression

An interesting avenue of research involves identifying the mechanism for the association between the two coding region mutations (SNP2 and 3) and growth traits in beef cattle. The coding region is the key region for gene function; the polymorphisms in this region can alter gene expression. To further confirm the effect of *ZBED6* overexpression on the regulation of *IGF2* expression, we used qPCR to detect the amount of *ZBED6* and *IGF2* mRNA expression in the C2C12 cells from overexpressed wild-type Hap-CA and mutant-type Hap-GG *ZBED6*. As shown in Figure 5B, the *ZBED6* mRNA expression level was 2.8284±0.289 in the wild-type (pcDNA3.1^+^ -Hap-CA) and 1.8532±0.248 in the mutant-type (pcDNA3.1^+^ -Hap-GG), and the *ZBED6* levels were low in mutant-type *ZBED6*-overexpression cells (*P* < 0.05). The expression of *IGF2* mRNA was significantly upregulated by mutant-type (8.4561±0.386) and wild-type (4.6589±0.239) cells compared with the vector control.

## Discussion

### Expression Profile

In the present study, the expression profile results indicated that *ZBED6* and *IGF2* had the lowest expression level at FB and gradually increased in abundance during the three different developmental stages of the liver. In contrast, the highest expression levels occurred at FB and gradually decreased in abundance during the three different developmental stages in the other tissues and organs. However, the expression levels in the spleen did not change between 90 days FB and 3-day-old NB but significantly decreased in the 24-month-old AB spleen organ. The expression patterns and levels of the *ZBED6* and *IGF2* genes in all tested bovine tissues and organs suggests that these two genes may play an important role during development, cell proliferation and growth in placental mammals. The functional characterization of *ZBED6* shows that it may affect the expression of thousands of other genes in addition to *IGF2* that control fundamental biological processes [8]. Therefore, further research is needed to discover their regulatory functions.

### Bioinformatic Analyses Predict the Allele-Dependent Presence of Different TFBSs

Despite the importance of *ZBED6* in muscle development, little is known about its regulation. To identify the possible TFBS in the bovine *ZBED6* promoter, we analyzed the promoter sequence using web-based software. The results showed that there were many potential factors; several binding sites for transcription factors were predicted for the region containing the myocyte enhancer factor-2 (MEF2), *POU domain,* class *1*, *transcription* factor *1* (*PIT1*) and *MYOD. MEF2* transcription factors control muscle-specific and growth factor-inducible genes [21]. *MEF2* was originally identified as a transcription factor complex through promoter analysis of the muscle creatine kinase (mck) gene to identify nuclear factors interacting with the mck enhancer region during muscle differentiation [22]. *PIT1* is a pituitary-specific transcription factor responsible for pituitary development and hormone expression in mammals and the regulation of many genes, including the growth hormone gene [23]. *PIT1* was shown to be associated with the economically relevant traits of many livestock, such as porcine carcass traits [24], bovine milk yield and conformation traits [25] and milk performance traits [26]. MyoD is a muscle-specific transcription factor that belongs to a family of proteins known as myogenic regulatory factors (MRFs); it is a protein with a key role in regulating muscle differentiation. MyoD can activate downstream myogenic structural genes and myogenic conversion in many different cell types. MyoD also activates its own transcription and the transcription of myogenin, another member of the MyoD gene family [27]. Recent studies have demonstrated that MyoD initiates a feedforward regulation of skeletal muscle gene expression and predict that MyoD binds directly to many genes expressed during differentiation. MyoD binds on a genome-wide basis and has the ability to broadly alter the epigenome in myoblasts and myotubes [28].

The presence of all of the described transcription factor binding sites in the bovine *ZBED6* promoter indicates that it is under a high level of transcriptional control by several transcription factors. Future research should address the interaction between all of these sites to provide a better understanding of *ZBED6* regulation and the myogenic process.

### Promoter Analysis of the *ZBED6* Gene

We carried out the deletion mutant analysis to demonstrate the effect of the transcription factors on the activity of the promoter. The results of a dual-luciferase reporter assay (Figure 4) showed that the full-length promoter fragment (P1) has a strong promoter activity (the empty reporter vector pGL3-base as control), indicating that the obtained promoter region was correct. P2 to P11 had promoter activity similar to or higher than the full-length promoter, showing that these regions contain transcription factor binding sites mediating positive regulation. Among them, the activity of P8 was significantly higher than that of other promoter fragments.

The *ZBED6* gene is located in the first intron of *zinc finger CCCH type containing 11A* (*ZC3H11A*), but the *ZBED6* protein has no significant sequence similarity to the ZC3H11A protein. Chromatin immunoprecipitation (ChIP) followed by high-throughput DNA sequencing (ChIP-Seq) revealed approximately 2,500 genomic regions bound by *ZBED6*, including the *IGF2* locus, and approximately 1,200 genes had at least one putative *ZBED6* binding site occurring within 5 kb of the TSS in C2C12 cells. The gene ontology analysis results suggest that *ZBED6* is an important regulator of development, cell proliferation and growth [8].

SNP1 is located in the 5’ flanking region of the *ZBED6* gene. Normally, transcription factors and *cis*-acting elements are located in this region, and these elements are the recognition sequences for transcription factors. Transcription factors regulate the start and efficiency of gene transcription by binding to *cis*-acting elements. Therefore, we speculated that mutations in the recognition sequences can change the binding between the transcription factors and *cis*-acting elements, thus regulating gene transcription and affecting the related signaling pathways. To test this inference, a previous study performed an association analysis between the genotype and bovine growth traits, and the results revealed that the cattle with genotype SNP1-AA could be selected to obtain greater growth traits [29]. We hypothesize that the mutations in the flanking region of the *ZBED6* gene may change the *cis*-acting elements associated with this gene and thus the transcription of this gene. The mutations in the promoter of the bovine lactoferrin (LTF) gene lead to different basal transcriptional activities that are associated with the lactoferrin levels in milk [30].

In the current study, through conjoint analysis of the data in Table S5 and S6, we found that the mutant-type individuals (-826A) (Figure 3) with the recognition sequences of the promyelocytic leukemia zinc-finger (PLZF) transcription factors (Table S6, Figure 3) exhibited better performance than the individuals with wild-type SNP1-G. The PLZF belongs to the POK (POZ and Krüppel) family of transcriptional repressors. This domain, common to several zinc finger-containing transcription factors, mediates protein-protein interactions and allows POZ domain proteins to participate in various differentiation pathways, including hematopoiesis, adipogenesis, osteoclastogenesis and muscle differentiation [31]. Therefore, we predict that individuals with mutant-type (-826A) in the recognition sequences of *cis*-acting elements may have better growth performance.

### Overexpression of *ZBED6* Is Associated with *IGF2* Expression

The results suggest that overexpression of mutant-type *ZBED6* significantly inhibited the expression level of the *ZBED6* gene. In contrast, the *IGF2* mRNA expression level was significantly up-regulated in C2C12 cells. This result is similar to the increased *IGF2* expression in skeletal muscle of pigs carrying the mutation at the *ZBED6* target site in *IGF2* gene [7]. *ZBED6* acts as a repressor of *IGF2* transcription in skeletal muscle myogenesis and development [8]. These studies suggested that *ZBED6* was a major regulator of *IGF2* expression in skeletal muscle but a repressor of the constitutive expression of *IGF2*.

## Conclusions

Three SNPs of the *ZBED6* gene exist in cattle population; these SNPs have some effects on *ZBED6* transcription activity and mRNA expression. Results obtained with the promoter analysis and gene expression data suggest that the mutant-type M-826A-pGL3 has the lowest promoter activity, the mutant-type Hap-AGG and pcDNA3.1^+^-Hap-GG significantly inhibited *ZBED6* expression and up-regulated *IGF2* mRNA and *ZBED6* negatively regulated the expression of the *IGF2* gene and thus may affect *IGF2* transcription in skeletal muscle myogenesis and development. However, we conclude that further research and validation of the various allelic effects, functional mechanisms and bioactivity are needed in an independent sample. These results may provide important insights into genes that are associated with the adaptation and specialization of beef cattle breeds in China.

## Supporting Information

Table S1 **Primer sets for PCR used for SNPs detected in bovine *ZBED6* gene.**
(DOC)

Table S2 **Primer used in qPCR and *ZBED6* overexpression analyses.**
(DOC)

Table S3 **Primer pairs used for creation of deletion mutation analyses in bovine *ZBED6* gene.**
(DOC)

Table S4 **Primer used in the 5’-RACE analyses.**
(DOC)

Table S5 **In silico analyses predict the allele-dependent presence of different TFBS in the wild-type ZBED6 promoter region.**
(XLS)

Table S6 **In silico analyses predict the allele-dependent presence of different TFBS in the mutant-type ZBED6 promoter region.**
(XLS)
