# Supplementary material for: A 5′- Regulatory Region and Two Coding Region Polymorphisms Modulate Promoter Activity and Gene Expression of the Growth Suppressor Gene ZBED6 in Cattle
Source: PLoS One. 2013 Nov 6;8(11):e79744. doi: 10.1371/journal.pone.0079744 (PMC3819241; doi:10.1371/journal.pone.0079744)
Supplement: Table S1 — Primer sets for PCR used for SNPs detected in bovine ZBED6 gene. (DOC) [file pone.0079744.s001.doc]

**Table S1.**

**Primer sets for PCR used for SNPs detected in bovine *ZBED6* gene.**

| SNPs **^1^** | Primer sequence (5’-3’) | Location **^2^** | AT (^o^C) **^3^** | SAF (bp) **^4^** |
| --- | --- | --- | --- | --- |
| SNP1 | F: GACACTTCCGTTCTCCTTGTG | nt-1123~nt-1103 | 61.8 | 1148 |
|  | R: GTACACTTAAGGTACATACACTCATC | nt +1~ nt +25 |  |  |
| SNP2-3 | F: CTGGAGGGCTATTTGTA | nt+438~nt+454 | 57.0 | 809 |
|  | R:TTGCCTGACTTATTTGAC | nt+1229~nt+1246 |  |  |

F: Forward primer; R: Reverse primer.

^1^ SNPs: SNP1: *ZBED6-*Promoter G-826A (ss#647298822); SNP2: *ZBED6-*Exon 1 C680G (ss#647298824); SNP3: *ZBED6*-Exon 1 A1043G (ss#647298826).

^2^ nt: nucleotide(s); relative to initiation codon ATG

^3^ AT=Annealing temperature.

^4^ SAF=Size of amplification fragment.
