# Supplementary material for: A 5′- Regulatory Region and Two Coding Region Polymorphisms Modulate Promoter Activity and Gene Expression of the Growth Suppressor Gene ZBED6 in Cattle
Source: PLoS One. 2013 Nov 6;8(11):e79744. doi: 10.1371/journal.pone.0079744 (PMC3819241; doi:10.1371/journal.pone.0079744)
Supplement: Table S2 — Primer used in qPCR and ZBED6 overexpression analyses. (DOC) [file pone.0079744.s002.doc]

**Table S2.**

**Primer used in qPCR and ZBED6 overexpression analyses.**

| Gene | Primer sequences (5’-3’) | AT (^o^C) **^2^** | SAF (bp) **^3^** |
| --- | --- | --- | --- |
| *ZBED6* | *Mouse*: F: CAAGACATCTGCAGTTTGGAATTT R: TGTCGTTGAAGTGTTGAAGTTCCTA | 60 | 135 |
|  | Bovine: F: GGAACAAGAGCCAAGAC; R: CCAATGGATGGGATGAG | 60 | 174 |
| *IGF2* | *Mouse*: F: CGTGGCATCGTGGAAGAGT; R: ACACGTCCCTCTCGGACTTG | 60 | 94 |
|  | *Bovine:* F: TCTGTGCGGCGGGGAGCTGGT; R: AGTCTCCAGCAGGGCCAGGTCG | 60 | 154 |
| *GAPDH* | *Mouse*: F: AGACAGCCGCATCTTCTTGT; R: TTCCCATTCTCAGCCTTGAC | 60 | 239 |
|  | *Bovine:* F: CGACTTCAACAGCGACACTCAC; R: CCCTGTTGCTGTAGCCAAATTC | 60 | 118 |
| *β-actin* | *Mouse*: F: CAGAGCAAGAGAGGCATCCTC; R: GTCCAGACGCAGGATGGCATG | 60 | 363 |
|  | *Bovine:* F: GTCATCACCATCGGCAATGAG; R: AATGCCGCAGGATTCCATG | 60 | 84 |
| pcDNA3.1^+^-*ZBED6* **^1^** | *Bovine:* F: **CGG**ggtaccGCCACCATGcaccaccaccaccaccacAGTGTATGTACCTTAAGTGTACC | 55.5 | 2943 |
|  | *Bovine:* R: **GC**tctagaTTAAGGTAATATTTCTTTTTCATTGC |  |  |

F: Forward primer; R: Reverse primer.

^1^ The primer used for constructing vectors pcDNA3.1^+^; squared nucleotides: which contains *Kpn* I (forward primer) and *Xba* I (reverse primer) restriction sites, as indicated by the lower-case letters; The protective base pairs “CGG” and “GC” were added in order to express in the 5' terminal of primer has also deliberately added black bold font. Underlined nucleotides: mark recognition site for the His-tag sequences;

^2^ AT=Annealing temperature.

^3^ SAF=Size of amplification fragment.
