# Supplementary material for: A 5′- Regulatory Region and Two Coding Region Polymorphisms Modulate Promoter Activity and Gene Expression of the Growth Suppressor Gene ZBED6 in Cattle
Source: PLoS One. 2013 Nov 6;8(11):e79744. doi: 10.1371/journal.pone.0079744 (PMC3819241; doi:10.1371/journal.pone.0079744)
Supplement: Table S3 — Primer pairs used for creation of deletion mutation analyses in bovine ZBED6 gene. (DOC) [file pone.0079744.s003.doc]

**Table S3.**

**Primer pairs used for creation of deletion mutation analyses in bovine *ZBED6* gene.**

| Primer name | Primer sequence (5’-3’) **^1^** | Location **^2^** | SAF (bp) **^3^** | Size (bp) **^4^** |
| --- | --- | --- | --- | --- |
| pGL3-P1 | F: **CGG** ggtaccGGGTGGAATCTTAGTAGGCAG | nt-3640~nt-3620 | 3665 | 3640 |
| pGL3-P2 | F: **CGG** ggtaccCTTGTAGGGCTGGTTG | nt-3327~nt-3312 | 3352 | 3327 |
| pGL3-P3 | F: **CGG** ggtaccGGTAAGCAACGGATGT | nt-2735~nt-2720 | 2760 | 2735 |
| pGL3-P4 | F: **CGG** ggtaccAAGATAAGGGATGCTGC | nt-2365~nt-2349 | 2390 | 2365 |
| pGL3-P5 | F: **CGG** ggtaccCTCAGTTCAGGGAGATACAA | nt-1904~nt-1885 | 1929 | 1904 |
| pGL3-P6 | F: **CGG** ggtaccTGCTCCTTTCCCAACCTTCTTCCCC | nt-1480~nt-1457 | 1505 | 1480 |
| pGL3-P7 | F: **CGG** ggtaccGACACTTCCGTTCTCCTTGTG | nt-1122~nt-1103 | 1147 | 1122 |
| pGL3-P8 | F: **CGG** ggtaccGCTTATATAGAGTCAAACTTAGGAG | nt-865~nt-842 | 890 | 865 |
| pGL3-P9 | F: **CGG** ggtaccGCCAAATTGCAGCCCTAAGTGGTG | nt-555~nt-533 | 580 | 555 |
| pGL3-P10 | F: **CGG** ggtaccTAAATTGGACTATTTGAATTCAG | nt-261~nt-240 | 286 | 261 |
| pGL3-P11 | F: **CGG** ggtaccTGGAGAAAAGGTAATGCAAG | nt-100~nt-82 | 125 | 100 |
| pGL3-P (1-11) | R: **GGA** agatctGTACACTTAAGGTACATACACTCATC | nt +1~ nt +25 | 0 | 0 |

F: Forward primer; R: Reverse primer.

^1^The primer used for constructing vectors pGL3-P (1-6), squared nucleotides: which contains *Kpn* I (forward primer) and *Bgl* II (reverse primer) restriction sites, as indicated by the lower-case letters. The protective base pairs “CGG” and “GGA” were added in order to express in the 5' terminal of primer has also deliberately added black bold font.

^2^ nt: nucleotide(s); relative to initiation codon ATG.

^3^ SAF=Size of amplification fragment.

^4^ The location and size of each 5’-deletion fragment is indicated to the left of each bar relative to the translation initiation “ATG” codon.
