# Supplementary material for: A 5′- Regulatory Region and Two Coding Region Polymorphisms Modulate Promoter Activity and Gene Expression of the Growth Suppressor Gene ZBED6 in Cattle
Source: PLoS One. 2013 Nov 6;8(11):e79744. doi: 10.1371/journal.pone.0079744 (PMC3819241; doi:10.1371/journal.pone.0079744)
Supplement: Table S4 — Primer used in the 5’-RACE analyses. (DOC) [file pone.0079744.s004.doc]

**Table S4.**

**Primer used in the 5’–RACE analyses.**

| Primer name | Primer sequence (5’-3’) | Location **^1^** |
| --- | --- | --- |
| 5’ -CDS | dT_25_ VN (N = A, C, G, or T; V = A, G, or C) |  |
| SMART II A | AAGCAGTGGTATCAACGCAGAGTACGCGGG |  |
| UPM | 5:1 mixture of Long (0.4 μM): Short (2 μM) |  |
|  | Long: CTAATACGACTCACTATAGCAAGCAGTGGTATCAACGCAGAGT |  |
|  | Short: CTAATACGACTCACTATAGC |  |
| NUPM | AAGCAGTGGTATCAACGCAGAGT |  |
| GSP1 | GAGGTGAGCACAACTCCACTGGCTCTAC | nt 2015~nt 2042 |
| GSP2 | CAGACGGTAACCTATAGTCAGGGGCCAC | nt 1324~nt 1351 |
| NGSP1 | CAGAGGTTACAAATAGCCCTCCAGG | nt 437~nt-461 |

^1^ nt: nucleotide(s); relative to initiation codon ATG.
